# Exploring configurations of social determinants for enhancing older adult health in China: an fuzzy-set qualitative comparative analysis based on 31 provinces in China

**DOI:** 10.3389/fpubh.2023.1255877

**Published:** 2023-10-03

**Authors:** Songbiao Zhang, Xining Wang, Huilin Wang

**Affiliations:** ^1^School of Business, Hunan University of Science and Technology, Xiangtan, China; ^2^Research Center for Regional High-quality Development, Xiangtan, China

**Keywords:** social determinants of health, older adult health, configurational effects, fsQCA, health policy

## Abstract

With China’s aging population on the rise, addressing population aging has become a national priority, particularly focusing on improving older adult health. This study employs the social determinants of health framework, considering China’s unique macro-social, economic, policy, healthcare, and family cultural factors, to develop a framework for understanding the social determinants of health for older adult in China. Using the fsQCA method and a configurational perspective, the complex relationship between social determinants of health and older adult health status is examined. The findings indicate that individual social determinants alone are insufficient for achieving high levels of older adult health. Instead, three configurations of social determinants have been identified as conducive to high older adult health: Economic Development-Environment – Cultural Dominant Type, Socio-Economic Development – Older Adult Security – Environment – Cultural Dominant Type, and Economic Development Dominant Type. These configurations offer diverse pathways for enhancing older adult health. Conversely, the study identifies two configurations associated with low older adult health levels, exhibiting an asymmetric relationship with the configurations resulting in high older adult health levels. Moreover, economic development consistently emerges as a core condition across all three configurations associated with high older adult health levels, while two configurations associated with low older adult health lack this core condition. These findings underscore the universal contribution of enhancing economic development to improving older adult health.

## 1. Introduction

Population aging is an inevitable and objective trend in the development of any country or region (1). According to the 7th National Population Census data in China in 2020, the proportion of the population aged 60 and above was 18.70%, and the proportion of the population aged 65 and above was 13.50% (2). Relevant departments predict that the aging process will further accelerate in the near future. With the deepening of population aging in China, a key measure to address this issue is to focus on the health of the older adult (3). As society and the economy continue to develop, people’s understanding of health has evolved from “the absence of disease or injury” to a broader perspective. The World Health Organization (WHO) defines health as “a state of complete physical, mental, and social well-being and not merely the absence of disease or infirmity (4).” In addition to this, research on factors influencing health has also advanced. Modern health concepts suggest that population health is influenced by various factors, including genetics, behaviors, and lifestyles, which have direct impacts on health (5). However, the WHO emphasizes that social determinants of health (SDH) play a fundamental role in determining people’s health and disease, affecting various stages of life, particularly during old age. The characteristics of SDH vary across different countries and periods, making them important entry points for policy interventions (6, 7).

Currently, research on the social factors influencing older adult health often focuses on single perspectives. For example, macro factors such as socioeconomic conditions (8), healthcare policies and systems (9), social security policies (10), and environmental factors (11), as well as micro factors such as individual income (12) and family support (13), have been analyzed using traditional regression analysis to examine the average effects of these factors on older adult health. However, older adult health is a complex phenomenon resulting from the interactions of internal and external factors (14). The potential interactions among various influencing factors can lead to different effects on older adult health. Moreover, the macro and micro factors influencing older adult health differ across provinces in China, and the combination of different factors also produces varying effects on older adult health. Therefore, it is crucial to identify an effective path that can generate high levels of older adult health, which provides important policy implications.

Based on a configurational perspective, this study employs the fuzzy-set Qualitative Comparative Analysis (fsQCA) method to analyze necessary and sufficient complex causal relationships between the social determinants of health and older adult health levels. The study aims to answer the following questions: (1) What macro and micro indicators should be included in a Chinese-specific framework for the social determinants of health? What are the weights of each factor? (2) Are the social determinants of health in China necessary conditions for achieving high levels of older adult health? (3) Which configurations of the social determinants of health can sufficiently generate high levels of older adult health? Which factors are universally present in configurations associated with high levels of older adult health? This study may contribute in the following ways: (1) Based on the theoretical framework of social determinants of health, we construct a Chinese-specific framework for the social determinants of older adult population health, filling the conceptual gap in the SDH framework for the older adult population in China and providing a new perspective for studying the influencing factors of older adult population health. (2) By using the fsQCA method and adopting a configurational perspective, we integrate antecedent conditions composed of socioeconomic development, policies, environment, and culture, and explore the relationship between different configurations and older adult health levels, offering new insights for the empirical study of social determinants of health and older adult health levels. (3) By introducing a configurational perspective to explore the complex impact of the social determinants of health on older adult health levels, this study has important theoretical and practical significance for investigating key factors and multiple paths to improve the health levels of the older adult population.

## 2. Literature review

### 2.1. Social determinants of health

Social Determinants of Health (SDH) refer to the environmental conditions in which people are born, live, learn, work, play, and age. These conditions have broad impacts on our health, functioning, and quality of life (15), and they are determined by the distribution of money, power, and resources at global, national, and local levels. Healthy China 2030 categorizes social determinants of health into five major groups: economic stability, educational opportunities and quality, access to healthcare and its quality, social and community context, and neighborhood and built environment. Research defines them as the primary factors that induce disease occurrence, beyond the factors that directly cause diseases, making them the “causes of causes” of diseases (16).

### 2.2. Social determinants of health and older adult health

#### 2.2.1. Socioeconomic development and older adult health

A series of research findings targeting the older adult population indicate that there is still a stable and continuous positive relationship between individuals’ physical health and socioeconomic status. Older adult individuals from privileged social classes exhibit better health conditions (17, 18). The key factor leading to this relationship is the access to “elastic resources” (19), which are resources that can help people avoid health risks or minimize the consequences of risks. These resources mainly include income, knowledge, power, prestige, and various interpersonal relationships within the social network (20). Some studies suggest that the impact of socioeconomic development on health is limited. Economic development in a region often leads to comprehensive improvements in residents’ living environment, social infrastructure, and the quality of public services, which have a universally positive spillover effect on health. However, as economic development reaches a certain level, this spillover effect gradually diminishes (referred to as the “ceiling effect”). Consequently, the relationship between economic development and residents’ health becomes less significant (21). However, considering the current situation in China, the influence of socioeconomic development on older adult health remains significant. Socioeconomic development is primarily reflected in macroeconomic variables such as *per capita* GDP (22, 23), employment opportunities (24), urbanization, and the proportion of the population in poverty (25).

#### 2.2.2. Policies and older adult health

Policies related to older adult health are closely intertwined with healthcare, medical services, and older adult care. They constitute an essential component of a country’s governance system. Brave man et al. highlighted that the healthcare system is a crucial health determinant significantly influenced by policies (26). Physical and mental health often deteriorate with age, and the aging population requires more healthcare services, leading to an increased overall demand for medical care (27). The vulnerability of the older adult population makes them the primary beneficiaries of healthcare services. The improvement of service systems can effectively meet the nursing requirements of the older adult population and enhance their health outcomes (28). Personal pension payments serve as a vital lever of social pension security. They are a crucial source of support for older adult living and play a strong positive role in promoting older adult health and reducing health inequalities (29, 30). Additionally, the improvement of social pension resources, such as the development of nursing homes and other infrastructure, also contributes to the promotion of older adult health (31).

#### 2.2.3. Environment and older adult health

Previous research has revealed a close relationship between natural and community environments and individuals’ health and survival (32). Older adult are more sensitive to the social and natural environments around them, and environmental deterioration has a more pronounced impact on their health and survival compared to younger adults (33). Rapid population aging, coupled with the ecological degradation resulting from rapid economic growth, significantly affects the quality of life for older individuals and their families. The relationship between community environment and older adult health has been extensively studied (34–36). The community environment reflects the physical infrastructure of a community, including green spaces, accessible roads, fitness facilities, and other elements (37).

#### 2.2.4. Culture and older adult health

With the socioeconomic development, the heterogeneity of China’s older adult population continues to increase (3). The expansion of higher education opportunities implemented in China since 1990 has facilitated an improvement in the overall educational attainment of the population. As cohorts progress, the heterogeneity in educational attainment among the older adult population is expected to further increase. Studies have shown that the disparity in health outcomes is closely related to differences in educational attainment (38). Family support, as a core component of informal care, has drawn attention from scholars regarding its influence on the health of older adult. Some studies suggest that support from family members contributes to improved health outcomes for older individuals (39).

**Figure 1:**
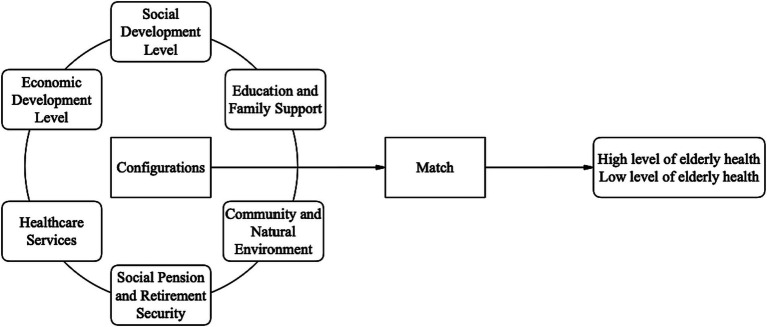
Theoretical framework of the influence of social determinants of health on older adult health.

In summary, previous research provides a foundation for understanding the relationship between social determinants of health and older adult health. This study adopts a configurational perspective and employs the fsQCA method to explore the diverse pathways through which social determinants of health at the provincial level promote improvements in older adult health. The study aims to answer how the combination of social determinants of health can be optimized to effectively enhance older adult health and achieve healthy aging. The theoretical model of this study is illustrated in Figure 1.

## 3. Research methodology and data

### 3.1. QCA method

The QCA (Qualitative Comparative Analysis) method is a case-oriented approach used to address the interdependencies and complexity of configurational phenomena. It was proposed by Ragin (40). In this study, the fsQCA (fuzzy-set Qualitative Comparative Analysis) method is employed, which is based on set theory and Boolean algebra. It analyzes the combinations of necessary and sufficient conditions that lead to specific outcomes.

The fsQCA method is chosen to explore the complex causal mechanisms underlying the level of older adult health for several reasons. Firstly, previous research has shown that older adult health is a complex phenomenon influenced by the interaction of various internal and external factors. Therefore, to understand the pathways to improve older adult health, it is insufficient to rely solely on statistical analyses of independent or pairwise interactions among the explanatory variables. The fsQCA method, starting from a holistic perspective, addresses this limitation by analyzing the complex causal relationships among multiple factors. Secondly, the high level of older adult health outcomes suggests the existence of multiple equivalent causal chains for improving older adult health. The fsQCA method helps researchers identify configurations of explanatory factors that lead to equivalent outcomes, allowing for an understanding of differentiated patterns in improving older adult health under different conditions. It further facilitates the discussion of complementary and substitutive relationships among the social determinants of health. Thirdly, fsQCA is well-suited to address asymmetric problems, where the causes of high older adult health levels are not necessarily the opposite of the causes of low older adult health levels. Finally, the fsQCA method is applicable to analyzing medium-sized samples ranging from 10 to 15–50 cases. In this study, the sample consists of 31 provincial regions, which falls within the medium-sized sample range.

### 3.2. Data

#### 3.2.1. Data sources

The study uses provincial regions in China as the basic spatial units (excluding Taiwan, Hong Kong, and Macau). The self-rated health data of the older adult population primarily come from the Seventh National Population Census. Data on the social determinants influencing older adult health levels are mainly sourced from the “China Statistical Yearbook,” “China Health Statistics Yearbook,” and the 2020 Seventh National Population Census.

#### 3.2.2. Variable selection and calibration

##### 3.2.2.1. Outcome variable

Self-rated health refers to the subjective self-assessment of an individual’s health status. It is a comprehensive evaluation of an individual’s health based on subjective feelings and objective symptoms. It can reflect aspects of health that objective health indicators may not capture. Self-rated health has been widely used in research to reveal the relationships between the physiological, psychological, social adaptation, and life satisfaction of older adults (41). It also has robust predictive power for cancer, cardiovascular diseases, mortality rates, and functional decline. Thus, self-rated health, as a multidimensional health indicator, not only reveals the current health status but also has predictive value for future health (42). Since 2005, self-rated health of the older adult population has become a key health indicator in the national population census. In the data from the Seventh National Population Census, a self-rated health status question was included for respondents aged 60 and above. The options for respondents to choose from include “healthy,” “basically healthy,” “unhealthy but can take care of oneself,” and “unable to take care of oneself.” In this study, “healthy” and “basically healthy” are defined as indicators of older adult self-rated health, and the proportion of older adult individuals with self-rated health is used as a measure of provincial older adult health levels.

##### 3.2.2.2. Explanatory variables

Based on the theoretical model of social determinants of health for the older adult constructed in the previous text, the six social influencing factors of health are evaluated through a three-level indicator weighting process, with weights determined based on relevant literature and data characteristics as follows:

1. Social Development Level: This factor mainly encompasses the degree of attracting immigrants and the level of aging (43). Hence, the economic development level is calculated by weighing two relative indicators: net migration rate (50%) and older adult dependency ratio (50%).
2. Economic Development Level: Referring to the practice in most literature (44), the *per capita* disposable income is chosen to represent the economic development status.
3. Health and Medical Services: This factor primarily includes the accessibility of health and medical resources. It is calculated by weighing two indicators: hospital beds per 10,000 people (50%) and healthcare personnel per 10,000 people (50%).
4. Social Pension Security: This factor mainly reflects the level of pension benefits (45). It is represented by the *per capita* pension level.
5. Community and Natural Environment: This factor encompasses the conditions of the community and natural environment. It is calculated by weighing two indicators: *per capita* green space area (50%) and *per capita* healthcare facility ownership (50%).
6. Education and Family Support: This factor includes the regional education level and the level of family support for the older adult. It is calculated by weighing two indicators: percentage of people with high school education and above (50%) and family support rate (50%) (Table 1).

**Table 1:** Indicator system of social determinants of older adult health in China.

Primary indicators	Secondary indicators	Tertiary indicators	Measurement methods
Socio-economic development factors	Social development level	Net population inflow rate	(Resident Population – Registered Population) divided by the registered population
		Older adult dependency ratio	Population aged 65 and above divided by the population aged 15–64
	Economic development level	*Per Capita* disposable income	Total disposable income of all residents divided by the resident population
Policy factors	Health and medical services	Number of medical and health institution beds per 10,000 people	Number of beds in medical and health institutions divided by the population, multiplied by 10,000
		Number of health technical personnel per 10,000 people	Number of health technical personnel divided by the population, multiplied by 10,000
	Social pension security	Average level of old-age pension	Average pension amount per person multiplied by the percentage of the population receiving pensions
Environmental factors	Community and natural environment	*Per Capita* green space area	Urban public green space area divided by the urban non-agricultural population
		*Per Capita* ownership of health facilities	(Number of community service institutions + pharmacies) divided by the resident population
Cultural Factors	Education and family support	Percentage of high school education and above	Number of individuals with high school education and above divided by the total population aged 15 and above
		Family support rate	Number of older adult people relying on family support divided by the population aged 60 and above

##### 3.2.2.3. Calibration

Before conducting the necessity and sufficiency analysis, it is necessary to calibrate the causal factors and the outcome. In this study, the direct calibration method of fuzzy sets is used to calibrate each variable. This method involves specifying values on a certain distance scale, where the three endpoints of the scale form a fuzzy set standard: completely non-membership, crossover point, and complete membership (46). Referring to previous research and combining empirical knowledge (47), this study selects the median, 5th percentile, and 95th percentile of each variable as the crossover point, complete non-membership, and complete membership threshold, respectively. Additionally, to avoid the problem of configuration membership exactly equal to 0.50 for the cases of causal factors, a constant of 0.001 is subtracted from the 0.5 membership degree in this study (48). The calibration results of each variable, along with descriptive statistical analysis, are presented in Table 2.

**Table 2:** Calibration results and descriptive statistical analysis.

Conditions and outcome	Fuzzy set calibration			Descriptive analysis			
	Complete membership	Crossover point	Completely non-membership	Mean	Standard deviation	Minimum value	Maximum value
Social development level	0.30	0.08	0.04	0.11	0.09	0.01	0.45
Economic development level	60915.50	27881.00	21769.50	32086.35	12455.12	20335.00	72232.00
Health and medical services	80.20	71.85	58.03	71.15	7.17	55.40	92.00
Social pension security	42412.81	19032.96	11859.63	22099.97	10378.26	11187.00	53412.00
Community and natural environment	12.67	8.71	7.06	9.30	1.80	5.83	13.21
Education and family support	0.40	0.35	0.28	0.34	0.04	0.26	0.43
Proportion of self-assessed health among the older adult	0.91	0.86	0.81	0.86	0.04	0.77	0.91

## 4. Results

### 4.1. Necessary analysis of individual conditions

In this study, fsQCA 3.0 was used to perform a necessary test on each condition, and the test results are shown in Table 3. It can be inferred that there are no individual causal conditions with consistency scores higher than 0.9, indicating that there are no single factors that have significant explanatory power in determining high or low levels of older adult health.

**Table 3:** Necessary test of individual conditions using fsQCA.

Condition variables	Determinant variables	Result variable			
		High level of older adult health		Low level of older adult health	
		Consistency	Coverage	Consistency	Coverage
X1	High level of social development	0.67522	0.74946	0.53751	0.56302
~X1	Low level of social development	0.60631	0.58144	0.76081	0.68853
X2	High level of economic development	0.73848	0.86368	0.46030	0.50803
~X2	Low level of economic development	0.57935	0.53217	0.87649	0.75978
X3	High level of healthcare services	0.60750	0.61288	0.68500	0.65216
~X3	Low level of healthcare services	0.65521	0.68791	0.59338	0.58791
X4	High level of social pension security	0.53220	0.60460	0.60800	0.65183
~X4	Low level of social pension security	0.69352	0.65214	0.63119	0.56011
X5	High level of community and natural environment	0.70537	0.70759	0.63777	0.60375
~X5	Low level of community and natural environment	0.60499	0.63896	0.69112	0.68883
X6	High level of education and family support	0.67772	0.69650	0.57936	0.56189
~X6	Low level of education and family support	0.57370	0.59105	0.68706	0.66798

### 4.2. Sufficiency analysis of configuration of conditions

Based on the necessary condition analysis, the fuzzy set truth table analysis procedure was used to incorporate the different causal conditions. Since this study had a sample size of 31, which falls within the range of small to medium-sized samples, a frequency threshold of 1 was determined. Additionally, to ensure a balanced configuration of 0 s and 1 s in the truth table, the original consistency threshold was set to 0.80, based on existing research. Finally, to avoid potential issues with contradictory configurations, the PRI consistency threshold was set to 0.70.

The fsQCA 3.0 software was run, and it produced complex solutions (without using logical remainders), intermediate solutions (utilizing logical remainders consistent with theory and practice), and parsimonious solutions (using all possible logical remainders that help simplify the configuration). Following existing research (47), the intermediate solution was employed, supplemented by the parsimonious solution to distinguish core conditions from peripheral conditions. The presence of a causal condition in the configuration is represented by “•,” while the absence is represented by “°.” In the configuration, a larger circle denotes a core condition, a smaller circle denotes a peripheral condition, and an empty space indicates that the presence or absence of the causal condition is not crucial for the outcome. The analysis results are presented in Table 4.

**Table 4:** Configuration for achieving high/low levels of older adult health.

Causal conditions	High level of older adult health			Low level of older adult health	
	H1a	H1b	H3	L1	L2
Level of social development	-	•	•	-	⊗
Level of economic development	●	●	●	Ⓧ	Ⓧ
Healthcare and medical services	⊗	-	Ⓧ	-	-
Social pension and older adult care security	⊗	•	•	●	●
Community and natural environment	●	●	Ⓧ	Ⓧ	Ⓧ
Education and family support	●	●	⊗	⊗	-
Consistency	0.9115	0.9234	0.9201	0.9375	0.9400
Original coverage	0.3361	0.2872	0.2526	0.3987	0.4266
Unique coverage	0.1335	0.0840	0.1078	0.0499	0.0778
Overall consistency	0.9223			0.9397	
Overall coverage	0.5284			0.4765	

● indicates the presence of a core condition; ⊗ indicates the absence of a core condition; • indicates the presence of a marginal condition; Ⓧ indicates the absence of a marginal condition; − indicates that the condition can either be present or absent without affecting the accuracy of the pathway.

Based on the analysis results, there are three configurations that generate high levels of older adult health: H1a, H1b, and H3. The consistency scores for these configurations are 0.9115, 0.9234, and 0.9201, respectively, all of which are greater than 0.9. This indicates that all the conditions are sufficient for generating high levels of older adult health. The overall consistency of the solutions is 0.9223, further confirming that the three configurations are sufficient conditions for high older adult health. The overall coverage of the solutions is 0.5287, indicating that the obtained configurations explain the main reasons for generating high levels of older adult health.

Similarly, there are three configurations that result in low levels of older adult health: L1 and L2. Their consistency scores are 0.9397 (≥0.90), and the coverage is 0.4765, demonstrating that these configurations are sufficient conditions for the outcome and explain approximately 48% of the reasons for low levels of older adult health. In the following analysis, we will examine each configuration in detail, separately for high and low levels of older adult health.

#### 4.2.1. Sufficiency analysis of high older adult health level

Horizontal analysis of various configurations (H1a, H1b, H2) for high older adult health levels reveals three patterns (47): Economic Development – Environment – Cultural Dominant Type, Socio-Economic Development – Older Adult Security – Environment – Cultural Dominant Type, and Economic Development Dominant Type.

Economic Development – Environment – Cultural Dominant Type (H1a): This configuration indicates that high economic development level, favorable natural and social environments, and good education and family support are core conditions. Even with relatively lower health and medical services and social pension security, high older adult health levels can still be achieved. The explanatory sample coverage rate is 0.3365, and the unique coverage rate is 0.1335, both being the highest among the three configurations. Typical provinces falling into this category include Tianjin and Zhejiang, which are situated in the economically advanced eastern coastal regions. These provinces have a leading economic development level, relatively well-built community healthcare facilities, and a higher average level of education among the older adult. Additionally, a substantial proportion of the older adult population receives support from their families.

Socio-Economic Development – Older Adult Security – Environment – Cultural Dominant Type (H1b): This configuration indicates that high economic development level, favorable natural and social environments, and good education and family support are core conditions. The high levels of social development and social pension security complement each other, enabling the achievement of high older adult health levels even in the absence of excellent health and medical services. H1b configuration exhibits symbiotic characteristics, where society, economy, policy, and culture benefit from each other’s existence, leading to a synergistic and mutually reinforcing relationship. Representative provinces falling into this category include Beijing, Shanghai, and Guangdong. Apart from their high socio-economic development levels, these provinces have more comprehensive social pension security policies and rich family caregiving cultures. Specifically, the social pension system includes substantial individual pension benefits, and family members provide considerable financial support. The increased income is then invested in the older adult’s health, leading to improved satisfaction and overall health levels.

Economic Development Dominant Type (H2): This configuration highlights high economic development level as the core condition, with high socio-economic development, low health and medical services, high social pension security, low community and natural environment, and low education and family support as the marginal conditions. It indicates that in areas where sufficient medical resources, favorable environment, and cultural conditions are lacking, high older adult health levels can still be achieved as long as the socio-economic development level is high and individual pension benefits are substantial. Specifically, higher *per capita* disposable income positively influences disease incidence and mortality rates, mental health, and subjective self-rated health among the older adult. Additionally, individual pension benefits play a vital role in preventing older adult poverty, thereby enhancing personal well-being and life satisfaction.

#### 4.2.2. Sufficiency analysis of low older adult health level

In order to examine causal asymmetry, this study analyzed the social determinants of health configurations that lead to low older adult health levels and identified three configurations associated with low older adult health. Firstly, configuration L1 reveals that in settings lacking high economic development, favorable community and natural environments, and education and family support, even with higher levels of social pension security, the older adult’s health level remains low. Representative provinces falling into this category include Tibet, Inner Mongolia, Xinjiang, and other western regions. These provinces experience relatively underdeveloped social development, with a significant outflow of young labor force resulting in a large population of older adult left behind, and the overall education level is relatively low. Despite substantial financial subsidies from the central government for social pension insurance, such as full government subsidies for urban and rural residents’ basic pension insurance in the western regions and increasing subsidies for the New Rural Cooperative Medical Care in the central and western regions, these measures cannot fundamentally improve the low health status of the older adult population. Secondly, configuration L2 indicates that in settings lacking high socio-economic development and favorable community and natural environments, regardless of the levels of health and medical services and education and family support, the older adult’s health level remains low. This demonstrates the significant impact of economic development and community and natural environments on older adult health.

### 4.3. Robustness test

This study conducted a robustness test on the configurations of social determinants that lead to high older adult health levels. QCA is a set-theoretic method that considers slight changes in operations as robust when the subset relations among the results do not alter the substantive interpretation of the research findings. Drawing on previous studies (49), this study employed the technique of changing the critical values for outcome variables and necessary conditions. Specifically, the completely non-membership and completely membership critical values were changed to the 10th percentile and 90th percentile, respectively. The consistency threshold and case frequency remained unchanged. The resulting consistency and coverage showed minimal variations, but the configuration pathways remained largely consistent. The results are presented in Table 5. The aforementioned test demonstrates that the research findings in this study possess good robustness.

**Table 5:** Robustness test.

Causal conditions	High proportion of older adult self-rated health		
	H1	H2	H3
Level of social development	•	•	•
Level of economic development	●	●	●
Healthcare and medical services	⊗	-	Ⓧ
Social pension and older adult care security	-	•	•
Community and natural environment	•	•	Ⓧ
Education and family support	●	●	⊗
Consistency	0.9344	0.9472	0.9510
Original coverage	0.5856	0.2586	0.2066
Unique coverage	0.0607	0.0802	0.0907
Overall consistency	0.9477		
Overall coverage	0.5109		

● indicates the presence of a core condition; ⊗ indicates the absence of a core condition; • indicates the presence of a marginal condition; Ⓧ indicates the absence of a marginal condition; − indicates that the condition can either be present or absent without affecting the accuracy of the pathway.

## 5. Discussion

### 5.1. Theoretical contributions

Firstly, this study builds upon the existing social determinants of health (SDH) theoretical framework and integrates China’s unique national conditions to construct an analytical framework that synergistically drives the health of the older adult at four macro-level elements: socio-economic development, policies, environment, and culture. This framework incorporates multiple conditional factors and derives six social determinants of health, providing robust theoretical support for subsequent empirical analysis by localizing the SDH theory to the older adult population in China.

Secondly, based on a configurational perspective, this study empirically examines the necessity and sufficiency of six key factors—socio-economic development, health care services, older adult social security, community and natural environment, education, and family support—for older adult health. It departs from the traditional regression analysis that selects a single variable to examine older adult health and overlooks the interdependencies among the predictors. This study serves as a valuable complement to existing literature in terms of methods and perspectives.

### 5.2. Practical significance

Based on the research on the relationship between social determinants of health and the level of older adult health, this study reveals that a single social determinant is insufficient to promote high levels of older adult health. Different provinces may have different paths to enhancing older adult health due to varying levels of socio-economic development, healthcare and social security policies, etc. Therefore, it is essential to optimize the combination of social determinants of health to promote the improvement of older adult health levels. The practical implications of the study are as follows:

1. Economic development is of paramount importance in improving older adult health. All three configurations associated with high levels of older adult health include high levels of economic development. Furthermore, the two configurations associated with low levels of older adult health have core deficiencies in economic development. This suggests that high levels of economic development have a universal impact on high levels of older adult health. *Per capita* disposable income, as an important indicator of economic development, plays a significant role in health. Therefore, increasing the income level of the older adult is of great importance to their health. Based on the current Chinese national conditions, measures such as raising the retirement age to increase the income of individuals over 60 with labor capabilities, increasing the basic pension level for urban and rural residents to improve the income standard of insured older adult individuals, and raising the minimum living allowance level to eliminate absolute poverty among the older adult can be implemented. However, while improving the overall income level of the older adult, it is necessary to comprehensively improve the income distribution system and guard against health inequalities resulting from income disparities.
2. Both configurations H1a and H1b, representing high older adult health levels, exhibit high levels of community and natural environments and strong education and family support. This indicates that while emphasizing economic development, it is crucial to be cautious about the potential sacrifices in natural environments due to high population density and the potential overcrowding of older adult health facilities. Additionally, investing in education should not be overlooked. Ensuring equal access to education opportunities, increasing educational investment to promote higher education among the population, and improving educational quality are all measures that can enhance older adult health levels. These initiatives will contribute to the improvement of overall older adult health while promoting educational equity and well-being.
3. Policy formulation needs to optimize the social determinants of health to synergistically enhance older adult health. There are diverse development paths for high levels of older adult health in different provinces. Considering the different development stages and resource endowments of different provinces, it is not feasible to expect the same optimization measures to achieve improvements in older adult health levels across all provinces. Instead, a “leader-follower” approach should be adopted, with leaders continuously exploring effective paths to promote older adult health and followers catching up to stimulate further exploration by the leaders, ultimately finding effective configurational paths to enhance older adult health.

### 5.3. Limitations

This study has the following limitations: Firstly, although the theoretical framework is based on China’s unique macro and micro-environmental conditions and builds upon the SDH theoretical framework, the complexity of factors influencing health makes it difficult to include all relevant factors. In the future, as society progresses and develops, the model can incorporate more macro and micro-level factors that influence older adult health. Secondly, constrained by data availability, this study only focuses on analyzing the static relationship between social determinants of health and older adult health levels. In the future, with the accumulation of data from sources like the national census and the improvement of indicators for social determinants of health, researchers can further analyze how changes in social determinants of health dynamically impact changes in older adult health levels, and use TQCA for time-series configurational analysis. Lastly, this study combines quantitative analysis with qualitative analysis on case studies, which helps reveal the mechanisms behind the quantitative findings. However, large-sample QCA studies lack the depth and richness of qualitative analysis that individual case studies provide. Future research can conduct in-depth case studies on different types of older adult populations (e.g., with and without spouses, with and without pensions) to reveal effective paths for enhancing the health levels of different older adult groups based on social determinants of health.

## 6. Conclusion

The optimization of social determinants of health to promote the improvement of older adult health levels is a focus of research on aging. Socio-economic development, policies, environment, and culture all play important roles in older adult health levels. This study, based on the theoretical framework of social determinants of health, focuses on China’s 31 provincial regions and uses fsQCA to analyze the relationship between social determinants of health and older adult health levels from a configurational perspective.

Firstly, this study establishes a comprehensive evaluation index system for older adult health based on the theoretical framework of social determinants of health, taking into account China’s national conditions in terms of socio-economic development, policies, environment, and culture. Secondly, using fsQCA, this study examines the necessity of each factor and finds that a single social determinant of health does not constitute a necessary condition for high levels of older adult health. However, high levels of economic development play a relatively universal role in achieving high levels of older adult health. Thirdly, using the configurational perspective and the QCA method, this study identifies three configurations of social determinants of health that lead to high levels of older adult health. These configurations reflect the multiple pathways to achieving high levels of older adult health in different provinces. Finally, this study finds that all three configurations associated with high levels of older adult health include high levels of economic development, while all three configurations associated with low levels of older adult health include low levels of economic development, indicating the significant role of income levels among the older adult in health.

## Data availability statement

The datasets presented in this study can be found in online repositories. The names of the repository/repositories and accession number(s) can be found at: http://www.stats.gov.cn/sj/pcsj/rkpc/7rp/indexch.htm; https://data.stats.gov.cn/easyquery.htm?cn=C01.

## Ethics statement

Ethical approval was not required for the studies involving humans because this study utilizes publicly available secondary data. The studies were conducted in accordance with the local legislation and institutional requirements. Written informed consent for participation was not required from the participants or the participants’ legal guardians/next of kin in accordance with the national legislation and institutional requirements because This study utilizes publicly available secondary data.

## Author contributions

SZ: Conceptualization, Funding acquisition, Methodology, Writing – original draft, Writing – review & editing. XW: Methodology, Writing – original draft, Writing – review & editing. HW: Conceptualization, Project administration, Writing – original draft, Writing – review & editing.
